# Elevated Hemoglobin A1c and the Risk of Postoperative Complications in Elective Hand and Upper Extremity Surgery

**DOI:** 10.7759/cureus.48373

**Published:** 2023-11-06

**Authors:** Cameron T Cox, Stephen Sierra, Alec Egan, Desirae McKee, Brendan J MacKay

**Affiliations:** 1 Orthopedic Surgery, Texas Tech University Health Sciences Center, Lubbock, USA

**Keywords:** carpal tunnel release, elective upper extremity surgery, risk factors, complications, infection, hba1c, elective hand surgery, glycemic control, hemoglobin a1c

## Abstract

Background: Studies addressing the utility of hemoglobin A1c (HbA1c) levels in predicting surgical complications have reported mixed results. In practice, many surgeons use HbA1c cutoffs to determine a patient’s eligibility for elective surgery. The literature is especially limited in evaluating HbA1c as a risk factor in elective hand and upper extremity surgery. This study aims to evaluate the association of elevated HbA1c levels with the risk of postoperative complications in elective hand and upper extremity surgeries.

Methods: We performed a chart review of patients who underwent these elective operations performed by a single surgeon at a single institution. The outcomes of 930 surgeries were collected up to three months postoperatively, 334 of which had pre or postoperative HbA1c levels recorded. All 930 surgeries were evaluated for association between diabetes mellitus (DM) and complication rates using Fisher’s exact test, absolute risk, odds ratio, and regression analysis.

Results: DM alone was not associated with an increased risk of postoperative complications. In diabetic patients with both diagnosed DM and HbA1c > 10%, the absolute risk of complications was significantly higher. None of the other HbA1c categories (between 6.5% and 10%) were associated with significantly higher odds of complications in patients with diagnosed DM.

Conclusions: In the context of elective hand and upper extremity surgery, glycemic control (measured by HbA1c) should be included as an element of the surgical preparedness algorithm, rather than an independent disqualifying factor.

## Introduction

Historically, diabetic patients are known to have an increased risk of postoperative complications compared to the non-diabetic population. These complications include but are not limited to infections, impaired wound healing, cardiovascular events, venous thromboembolism, and mortality. Given that poor glycemic control has been identified as a significant risk factor, hemoglobin A1c (HbA1c) has been used as a biomarker to evaluate patients’ preparedness for surgery [1].

The American Diabetes Association recommends a target of HbA1c < 7% for diabetic patients [2], and some have suggested that surgery should not be undertaken in patients who fail to meet this goal [3]. While protocols vary by hospital and surgeon, recommendations for cutoffs in a variety of surgical settings have been reported between 7% and 9% [4-7]. Though many use these cutoffs as eligibility criteria for elective surgical interventions, the literature is unclear on the predictive value of HbA1c levels for postoperative complications.

One of the most common and concerning surgical complications, especially in patients with poor glycemic control, is infection. In 2018, Sharma et al. performed a prospective cohort study following diabetic and nondiabetic surgical hand and forearm infections over three years. In diabetic patients, the need for recurrent drainage was not significantly different between those with good versus poor baseline glycemic control (HbA1c > 9.0%). However, patients with poor inpatient glycemic control (average inpatient blood glucose of >180 mg/dL) had a significantly higher need for additional drainage [8]. Given that surgical infections are more difficult to resolve in patients with poor glycemic control, it is important to accurately assess predisposing factors that increase risk.

The correlation of HbA1c levels with postoperative complications has been documented in a variety of surgical procedures with mixed results. In a systematic review of non-diabetic patients, Karimian et al. analyzed the results of six observational studies. Four studies reported a significant correlation between preoperative HbA1c levels and postoperative complications. Only two of these four reported increased infection rates with elevated HbA1c, and two reported no significant difference [9]. None of these studies, however, investigated the value of HbA1c as a biomarker for increased risk in hand and upper extremity surgery.

In hand surgery, HbA1c data have been examined to determine the association between increased HbA1c levels and the development of trigger digit after carpal tunnel release (CTR). In 2014, Grandizio et al. evaluated 1,217 cases of CTR, 214 of which were performed on diabetic patients. Of the non-diabetic cases, 3% developed trigger digit within six months of CTR, and 4% within one year. Of diabetic patients, 8% developed trigger digit within six months and 10% within one year. Thus, diabetes was identified as a significant risk factor for developing trigger digit after CTR. Interestingly, no significant association was found between HbA1c level at the time of CTR and the likelihood of developing TD [10]. Aside from this study, we were unable to find an analysis of HbA1c levels in hand and upper extremity surgery in the literature.

Elective surgeries of the hand and upper extremities such as carpal and cubital tunnel release, arthroplasty, and tendon transfer have been shown to significantly improve function, pain, and quality of life for patients [11-14]. Physical activity has been shown to aid in the management of diabetes mellitus (DM), and the elective surgeries described above often give patients the ability to return to an active lifestyle [15]. Full use of the hands and arms is critical in maintaining the quality of life for these patients. In consideration of the expected improvement gained by operative treatment and the current paucity of data, we designed a study to assess the efficacy of HbA1c as a biomarker for the risk of postoperative complications.

## Materials and methods

Approval for this study was obtained from our Institutional Review Board (Texas Tech University Health Sciences Center, Lubbock/Odessa IRB; L20-027). Charts were collected for all patients who underwent common elective hand and/or upper extremity surgeries performed by a single fellowship-trained hand surgeon from 2012 to 2018 at a single hospital (Tables 1, 2). Patients with HbA1c levels recorded ≤ one year prior to surgery were categorized in the preoperative HbA1c group (Tables 3, 4). Patients with HbA1c recorded ≤ one year after surgery were placed in the postoperative HbA1c group (Tables 3, 4). While HbA1c is known to have a life span of only three months, multiple studies have shown that HbA1c levels are typically stable (<0.5% increase or decrease) over longer periods of time [16-19]. Analysis was performed using HbA1c at ≤ three months, ≤ six months, and ≤ one year preoperatively as well as ≤ one year postoperatively to allow for a complete view of the data.

**Table 1:** Breakdown of surgeries included by number performed and percentage of the entire cohort represented. DM: diabetes mellitus; MCP: metacarpophalangeal; PIP: proximal interphalangeal.

Procedure performed	n (%)
Trigger digit release	340 (36.6%)
Carpal tunnel release	318 (34.2%)
Cubital tunnel release	212 (22.8%)
Cyst excision	182 (19.6%)
Ligament reconstruction/tendon interposition	169 (18.2%)
Trapezoidectomy	85 (9.1%)
Compartment release	57 (6.1%)
Guyon’s canal release	31 (3.3%)
Nerve transposition	27 (2.9%)
Tenosynovitis	10 (1.1%)
MCP/PIP arthroplasty	6 (0.6%)
Radial tunnel release	3 (0.3%)
Styloidectomy	3 (0.3%)
Epicondylectomy	2 (0.2%)
Carpectomy	1 (0.1%)
Amputation	1 (0.1%)
Total number of procedures performed	1,447
Total number of surgeries	930
Mean age	52.8 years (range: 17-83)
Diagnosed DM	210 patients
Mean duration of DM	54.5 months (range: 1-286)
Diagnosed peripheral neuropathy	73 patients

**Table 2:** Number of procedures performed in a single surgery.

Number of procedures	Number of surgeries
1	n = 428 (46.0%)
2	n = 333 (35.8%)
3	n ­­= 112 (12.0%)
4	n ­­= 32 (3.4%)
5	n ­­= 17 (1.8%)
6	n ­­= 6 (0.6%)
7	n ­­= 1 (0.1%)
8	n ­­= 1 (0.1%)
Total number of surgeries performed	930
Mean age	52.8 (range: 17-83)
Race/ethnicity	N (%)
White/Caucasian	437 (47.0%)
Hispanic/Latino	380 (40.9%)
Black/African American	109 (11.7%)
Asian	4 (0.4%)
Diabetes mellitus diagnosed	210 patients
Peripheral neuropathy	73 patients

**Table 3:** Individual procedures with postoperative complications and HbA1c of each respective patient. HbA1c: hemoglobin A1c.

Procedure(s)	Complication	HbA1c
Ligament reconstruction and tendon interposition (thumb), complete trapeziectomy, carpal tunnel release.	Infection	5.8%
Ligament reconstruction and tendon interposition (thumb), complete trapeziectomy.	Infection	5.8%
Ulnar nerve subcutaneous transposition. Release of dorsal ulnar sensory nerve from scar tissue.	Infection	6.0%
Trigger release (thumb).	Infection	None
Trigger release (index finger).	Infection	12.8%
Trigger release (long finger).	Infection	5.2%
Trigger release (long finger). Carpal tunnel release.	Dehiscence (trigger release wound)	None
Ligament reconstruction/tendon interposition (thumb), complete trapeziectomy plus flexor carpi radialis donor.	Pain, swelling	12.6%
Ligament reconstruction/tendon interposition (thumb), complete trapeziectomy.	Pain	None
Ligament reconstruction/tendon interposition (thumb), complete trapeziectomy. Carpal tunnel release.	Pain	None
Ligament reconstruction/tendon interposition (thumb), complete trapeziectomy.	Triggering	6.5%
Carpal tunnel release. Trigger release (thumb, long, and ring fingers).	Triggering (index and small fingers)	9.3
Cubital tunnel release.	Numbness	None
Cubital tunnel release.	Numbness, weakness	None
Endoscopic cubital tunnel release.	Weakness	6.0%
Endoscopic cubital tunnel release, open carpal tunnel release.	Pain	5.1%
Endoscopic cubital tunnel release, open carpal tunnel release.	Weakness	7.6%
Ulnar nerve transposition with pronator fascial lengthening.	Pain	5.9%
Ulnar nerve transposition revision with pronator fascia lengthening and neurolysis.	Numbness	None
Trigger release (long finger).	Tenosynovitis	5.3%
Trigger release (small finger).	Pain, excessive scarring	8.9%
First dorsal extensor compartment release.	Pain	None

**Table 4:** HbA1c & DM – odds ratios for risk of postoperative complications divided by HbA1c cutoff points, preoperative conditions, and procedures performed. HbA1c: hemoglobin A1c; DM: diabetes mellitus.

≤3 months preoperative HbA1c (n = 75)									
HbA1c cutoffs	Number elevated		Absolute risk		Odds ratio	95% confidence interval			P-value
HbA1c > 6.5%	(n = 41)		4.9% (2/41)		0.53	(0.083-3.37)			0.411
HbA1c > 7%	(n = 37)		5.4% (2/37)		0.67	(0.11-4.24)			0.513
HbA1c > 8%	(n = 23)		4.3% (1/23)		0.55	(0.06-5.17)			0.511
HbA1c > 9%	(n = 14)		7.1% (1/14)		1.01	(0.11-10.6)			0.655
HbA1c > 10%	(n = 7)		14.3% (1/7)		2.67	(0.26-27.8)			0.396
≤6 months preoperative HbA1c (n = 125)									
HbA1c cutoffs	Number elevated		Absolute risk		Odds ratio	95% confidence interval			P-Value
HbA1c > 6.5%	(n = 63)		3.2% (2/63)		0.65	(0.10-4.00)			0.492
HbA1c > 7%	(n = 51)		3.9% (2/51)		0.97	(0.16-6.00)			0.671
HbA1c > 8%	(n = 30)		3.3% (1/30)		0.78	(0.08-7.30)			0.654
HbA1c > 9%	(n = 16)		6.3% (1/16)		1.75	(0.18-16.7)			0.502
HbA1c > 10%	(n = 7)		14.3% (1/7)		4.75	(0.46-49.3)			0.254
≤1-year preoperative HbA1c (n = 204)									
HbA1c cutoffs		Number elevated	Absolute risk	Odds ratio			95% confidence interval	P-value	
HbA1c > 6.5%		(n = 94)	5.3% (5/94)	1.49			(0.39-5.71)	0.402	
HbA1c > 7%		(n = 74)	6.8% (5/74)	2.28			(0.59-8.78)	0.189	
HbA1c > 8%		(n = 48)	6.3% (3/48)	1.67			(0.40-6.93)	0.357	
HbA1c > 9%		(n = 24)	8.3% (2/24)	2.25			(0.44-11.5)	0.286	
HbA1c > 10%		(n = 12)	16.7% (2/12)	5.29			(0.97-28.8)	0.091	
≤1-year postoperative HbA1c (n = 67)									
HbA1c cutoffs		Number elevated	Absolute risk	Odds ratio			95% confidence interval	P-value	
HbA1c > 6.5%		(n = 21)	9.5% (2/21)	1.51			(0.23-9.78)	0.503	
HbA1c > 7%		(n = 16)	12.5% (2/16)	2.29			(0.35-15.1)	0.343	
HbA1c > 8%		(n = 12)	8.3% (1/12)	1.16			(0.12-11.4)	0.640	
HbA1c > 9%		(n = 7)	0.0% (0/7)	1.13			(1.03-1.23)	0.566	
HbA1c > 10%		(n = 1)	0.0% (0/1)	1.02			(0.99-1.05)	0.925	
All HbA1c (n = 271)									
HbA1c cutoffs		Number elevated	Absolute risk	Odds ratio			95% confidence interval	P-value	
HbA1c > 6.5%		(n = 116)	5.2% (6/116)	1.36			(0.43-4.31)	0.410	
HbA1c > 7%		(n = 87)	5.7% (5/87)	1.54			(0.48-5.00)	0.331	
HbA1c > 8%		(n = 58)	5.2% (3/58)	1.24			(0.32-4.72)	0.493	
HbA1c > 9%		(n = 30)	6.7% (2/30)	1.65			(1.34-7.92)	0.392	
HbA1c > 10%		(n = 16)	12.5% (2/16)	3.50			(0.70-17.5)	0.153	
Preoperative glucose level (n = 801)									
Glucose (mg/L)		Number elevated	Absolute risk	Odds ratio			95% confidence interval	P-value	
>140 mg/L		(n = 228)	1.8% (6/73)	0.35			(0.12-1.00)	0.026	
Diabetes mellitus (DM)									
Diabetes diagnosis		Number diagnosed	Absolute risk	Odds ratio			95% confidence Interval	P-value	
DM		(n = 210)	4.3% (9/210)	1.97			(0.86-4.53)	0.087	
Peripheral neuropathy (PN)									
Neuropathy diagnosis		Number diagnosed	Absolute risk	Odds ratio			95% confidence interval	P-value	
PN		(n = 73)	8.2% (6/73)	2.12			(0.51-8.82)	0.240	
Procedure									
		Number performed	Absolute risk	Odds ratio			95% confidence interval	P-Value	
Trigger digit release		(n = 340)	2.1% (7/340)	0.81			(0.33-2.01)	0.649	
Carpal tunnel release		(n = 318)	1.6% (5/318)	0.57			(0.21-1.55)	0.268	
Cubital tunnel release		(n = 212)	2.4% (5/212)	1.00			(0.36-2.73)	0.994	
Ligament reconstruction/tendon interposition		(n = 169)	3.6% (6/169)	1.69			(0.65-4.38)	0.281	
Trapezoidectomy		(n = 85)	3.6% (5/85)	2.92			(1.05-8.12)	0.040	
Compartment release		(n = 57)	1.8% (1/57)	0.73			(0.10-5.52)	0.760	
Nerve transposition		(n = 27)	11.1% (3/27)	5.28			(1.47-18.9)	0.011	

Follow-up clinic notes, diagnoses, and medications were evaluated three months postoperatively for the incidence of postoperative complications. Complications in our cohort were defined as infection, dehiscence, and unimproved or worsening of preoperative symptoms. Comorbidities, including diagnosed DM, peripheral neuropathy (PN), and macrovascular disease (coronary, cerebrovascular, or peripheral), were recorded.

HbA1c values were analyzed using cutoffs (>6.5%, 7%, 8%, 9%, or 10%) and categories (6.51-7%, 7.01-8%, 8.01-9%, and 9.01-10%). The absolute risk, odds ratios, and Fisher’s exact tests were calculated to evaluate the risk of postoperative complications in each group (Tables 3, 4). Presence DM, PN, elevated preoperative glucose (>140 mg/L), and procedures performed were evaluated in the same manner (Tables 3, 4). Patients were then divided into diabetic and non-diabetic subcategories, and the same statistical analyses were applied (Table 5).

**Table 5:** HbA1c and DM – odds ratios for risk of postoperative complications, divided by sections of HbA1c levels. HbA1c: hemoglobin A1c; DM: diabetes mellitus.

≤ 1-year preoperative HbA1c (n = 204)					
HbA1c	Number elevated	Absolute risk	Odds ratio	95% confidence interval	P-value
6.51-7.00%	(n = 27)	3.7% (1/27)	1.10	(0.13-9.48)	0.636
7.01-8.00%	(n = 29)	3.4% (1/29)	1.01	(0.12-8.67)	0.664
8.01-9.00%	(n = 23)	0.0% (0/23)	1.13	(1.08-1.19)	0.427
9.01-10.00%	(n = 9)	0.0% (0/9)	1.05	(1.02-1.08)	0.726
> 10.0%	(n = 15)	13.3% (2/15)	5.66	(1.00-32.1)	0.086
≤1-year postoperative HbA1c (n = 67)					
HbA1c	Number elevated	Absolute risk	Odds ratio	95% confidence interval	P-value
6.51-7.00%	(n = 5)	0.0% (0/5)	1.10	(1.01-1.17)	0.670
7.01-8.00%	(n = 5)	20.0% (1/5)	3.63	(0.32-40.5)	0.330
8.01-9.00%	(n = 5)	20.0% (1/5)	3.63	(0.32-40.5)	0.330
9.01-10.00%	(n = 6)	0.0% (0/6)	1.12	(1.02-1.20)	0.616
> 10.0%	(n = 1)	0.0% (0/1)	1.02	(0.99-1.05)	0.925
All HbA1c (n = 271)					
HbA1c	Number elevated	Absolute risk	Odds ratio	95% confidence interval	P-value
6.51-7.00%	(n = 32)	3.1% (1/32)	0.67	(0.08-5.36)	0.575
7.01-8.00%	(n = 34)	5.8% (2/34)	1.42	(0.30-6.77)	0.458
8.01-9.00%	(n = 28)	3.6% (1/28)	0.78	(0.10-6.29)	0.643
9.01-10.00%	(n = 15)	0.0% (0/15)	1.06	(1.03-1.09)	0.498
>10.0%	(n = 16)	12.5% (2/16)	3.50	(0.70-17.5)	0.153

Regression analysis was also performed to assess the degree of correlation between the incidence of elevated HbA1c, DM, duration of DM (time between official diagnosis to surgery), and macrovascular disease with postoperative complications (Table 6). Pearson’s regression coefficient was calculated to determine the relationship between elevated (>140 mg/L) preoperative glucose and the occurrence of postoperative complications.

**Table 6:** Breakdown of odds ratios for postoperative complications in diabetic versus non-diabetic patients. ^a^ None of the non-diabetic patients had preoperative HbA1c levels of 9.01-10% or postoperative HbA1c levels of 7.01-9% or >10%. HbA1c: hemoglobin A1c.

Diabetic patients - preoperative HbA1c (n = 106)					
HbA1c	Number elevated	Absolute risk	Odds ratio	95% confidence interval	P-value
6.51-7.00%	(n = 22)	0.0% (0/22)	1.28	(1.15-1.41)	0.388
7.01-8.00%	(n = 25)	4.0% (1/25)	1.08	(0.11-10.9)	0.665
8.01-9.00%	(n = 19)	0.0% (0/19)	1.23	(1.12-1.35)	0.448
9.01-10.00%	(n = 9)	0.0% (0/9)	1.10	(1.03-1.17)	0.697
>10.0%	(n = 15)	20.0% (3/15)	22.5	(2.16-234.0)	0.009
Diabetic patients - postoperative HbA1c (n = 41)					
HbA1c	Number elevated	Absolute risk	Odds ratio	95% confidence interval	P-value
6.51-7.00%	(n = 9)	0.0% (0/9)	1.32	(1.10-1.59)	0.355
7.01-8.00%	(n = 6)	16.7% (1/6)	2.13	(0.18-24.8)	0.483
8.01-9.00%	(n = 7)	14.3% (1/7)	1.72	(0.15-19.5)	0.542
9.01-10.00%	(n = 12)	8.3% (1/12)	0.79	(0.07-8.43)	0.668
>10.0%	(n = 3)	0.0% (0/3)	1.09	(0.99-1.20)	0.729
Non-diabetic patients - preoperative HbA1c (n = 99)					
HbA1c	Number elevated	Absolute risk	Odds ratio	95% confidence interval	P-value
6.51-7.00%	(n = 5)	20.0% (1/5)	2.69	(0.27-27.0)	0.386
7.01-8.00%	(n = 4)	0.0% (0/4)	1.05	(1.00-1.09)	0.679
8.01-9.00%	(n = 4)	25.0% (1/4)	3.63	(0.34-39.0)	0.321
9.01-10.00%^a^	-	-	-	-	-
>10.0%^a^	-	-	-	-	-
Non-diabetic patients - postoperative HbA1c (n = 89)					
HbA1c	Number elevated	Absolute risk	Odds ratio	95% confidence interval	P-value
6.51-7.00%	(n = 2)	0.0% (0/2)	1.02	(0.99-1.06)	0.890
7.01-8.00%^a^	-	-	-	-	-
8.01-9.00%^a^	-	-	-	-	-
9.01-10.00%	(n = 1)	0.0% (0/1)	1.01	(0.99-1.04)	0.944
>10.0%^a^	-	-	-	-	-

## Results

A total of 930 patients were included in our study. A total of 502 patients underwent more than one procedure (Table 1) in a single surgery, and a total of 1,447 procedures were performed (Table 2). Of the patients, 240 had preoperative HbA1c levels recorded ≤ one year prior to surgery with an average time from surgery of 4.5 months (SD: 8.6, range: 0-12). A total of 67 patients had postoperative HbA1c levels taken ≤ one year after surgery with an average time from surgery of 6.6 months (SD: 3.4, range: 1-12).

There were 22 complications at three months postoperatively in our entire cohort (n = 930). Infection accounted for 31.8% (7/22) of complications, and the remaining 68.2% (15/22) experienced no improvement or worsening of preoperative symptoms. A list of procedures paired with their respective postoperative complications can be found in Table 3.

In the preoperative HbA1c group, none of the HbA1c cutoffs were significantly associated with an increased risk of complication. Diagnosis of DM or PN also failed to increase risk (Tables 4, 5). For diabetic patients, the absolute risk of complications was significantly higher in patients with HbA1c greater than 10% (Table 6). Patients who underwent trapezoidectomy (OR: 2.92, p = 0.040) and/or nerve transposition (OR: 5.28, p = 0.011) had significantly increased odds of developing postoperative complications (Table 4). While other subsets showed an increased odds ratio with elevated HbA1c, none were statistically significant. Elevated preoperative glucose level (>140 mg/L) showed an inverse relationship with the risk of complications (Table 4). However, the correlation coefficient of preoperative glucose and the presence of postoperative complications (r = -0.072) did not indicate a significant relationship.

Regression analysis did not show a significant correlation between any HbA1c cutoff, DM, DM duration, or macrovascular disease with the incidence of postoperative complications (Table 7).

**Table 7:** HbA1c and DM - regression analysis ^a^ Beta = strength of correlation (beta = 1 for two identical sets of values). Negative values indicate a significant inverse relationship. ^b^ P ≤ 0.05 = significant correlation between predisposing factor and incidence of complications. HbA1c: hemoglobin A1c; DM: diabetes mellitus.

Correlation of HbA1c and DM with postoperative complications				
Predisposing	Unstandardized coefficients		Standardized coefficients	Significance
Factors	B	Std. error	Beta^a^	P-value^b^
>6.5%	-0.035	0.042	-0.090	-0.836
>7.0%	0.064	0.049	0.155	1.322
>8.0%	-0.025	0.050	-0.052	-0.489
>9.0%	-0.040	0.055	-0.069	-0.727
>10.0%	0.118	0.061	0.139	1.929
DM	0.003	0.029	0.007	0.097
Duration of DM	<0.001	<0.001	0.001	0.993
Macrovascular disease	<0.001	<0.001	<0.001	0.999

## Discussion

Principle among the concerns of operating on patients with high HbA1c levels is the risk of postoperative infection. HbA1c is a measure of glycemic control, which is frequently a concern for patients with DM. The hyperglycemic environment in diabetic patients can cause immune dysfunction by suppressing mechanisms needed to fight infection, including T-lymphocyte response, neutrophil function, and secretion of inflammatory cytokines [20]. These mechanisms lead to an increased incidence of infection in this population. While patients with poorly controlled DM generally have elevated HbA1c, high levels of HbA1c do not necessarily correlate with higher rates of postoperative infection.

A study by Endara et al. measured HbA1c and blood glucose levels five days before and after high-risk surgical closure. Univariate and multivariate analyses were performed to determine the correlation of elevated HbA1c levels (above 6.5% and 7.0%) with incidence of dehiscence, infection, and reoperation. Results showed that while dehiscence increased as HbA1c increased, there was no significant correlation between either cutoff and increased rates of infection and/or reoperation [4].

Given the variability in protocols and reported outcomes regarding HbA1c, recent studies have investigated additional comorbid conditions as potential risk factors. One prospective study evaluated 2,060 foot and ankle surgeries over a period of four years to determine the correlation of DM and PN with postoperative infection [21]. Investigators reported that PN significantly increased the odds ratio for surgical site infection, and the presence of DM did not significantly change the odds. They did find that in diabetic patients, HbA1c ≥ 8.0% was associated with a 2.7 times greater risk of infection [21].

In a separate study evaluating the relationship of surgical morbidity to glycemic control in elective foot and ankle surgery, Domek et al. reviewed 21,854 diabetic patients with HbA1c measurements < one year prior to elective foot and ankle surgery [22]. The postoperative complication rate was 3.2% from all causes combined. The most common complications were infection (42.3%), mechanical failure (33.4%), cardiovascular/pulmonary (18.4%), and wound healing (5.8%). The average HbA1c of a patient who had experienced a complication was 6.29% compared with 6.11% for a patient who had not experienced any complications. Results showed that for each 1% increase in HbA1c, the odds of developing a complication increased by 5%. Interestingly, the investigators concluded that HbA1c alone was insufficient to predict complication rates. While the odds of postoperative complication gradually increased with HbA1c, the presence of preoperative PN and/or comorbid conditions were stronger predictors of complications. Of note, there was no HbA1c cutoff point at which a steep increase in complication rate occurred [22].

HbA1c is not the only metric that has been questioned as a determinant for surgical candidacy. Hard cutoffs are often used for weight, BMI, and cigarette smoking as well [23]. Glycemic control, like these parameters, is seen by many as a modifiable factor. Surgeons may believe that they are only putting off surgery for these patients rather than withholding it entirely. Unfortunately, these so-called modifiable factors can be nearly impossible to actually modify, particularly in disadvantaged populations [23,24].

Even when resources are available, recommended levels of biomarkers such as HbA1c are notoriously difficult to achieve. In a study assessing the effects of literacy, numeracy, and medication adherence on glycemic control, Huang et al. found that medication adherence was only weakly correlated (rho correlation coefficient: 0.238) with glycemic control (HbA1c ≤ 7.0%) [25].

Setting hard cutoffs for HbA1c and other comorbid domains serves to deindividualize patients, and could lead to discriminatory practice against patients who would have tolerated the procedure well. It is well known that lower socioeconomic status (SES), as well as minority race and ethnicity, can negatively influence clinical outcomes such as glycemic control in diabetic patients [26]. Interestingly, there is no consensus on the influence of minority standing on the psychological profiles of these patients [27,28]. Psychological factors are known to influence outcomes following musculoskeletal surgery; however, patients are rarely denied operative treatment due to anxiety, depression, or pain catastrophizing [29].

Given the nature of blue-collar jobs, which often include manual labor, it is possible that low SES patients may benefit more from these operative interventions, as physical aptitude directly affects their livelihood. By allowing a single risk factor without consensus support in the literature to bar them from treatment, surgeons may unknowingly discriminate against these populations.

The present study is limited by the variable timing of HbA1c testing. Ideally, HbA1c measurements are taken < three months prior to surgery. HbA1c levels at ≤ six months and ≤ one year are indirect measures of preoperative glycemic control and are not intended to serve as a surrogate for HbA1c obtained within the three-month window. The majority of procedures included in our study have relatively low risk (e.g., trigger finger release, carpal tunnel release, and cyst excision). Future studies may better address procedures such as arthroplasties and tendon grafting - where infection and wound dehiscence are more likely to occur. Additionally, a small sample of patients in the higher HbA1c cutoff groups is prone to beta error and may be insufficient to draw conclusions regarding these patients in our cohort. Given the retrospective nature of this study, variables such as the duration of DM may not be precise. The date that DM is detected and documented in the electronic medical record (EMR) does not necessarily indicate the actual onset of DM.

## Conclusions

The results of our study add to a growing body of literature addressing the utility of HbA1c as a preoperative qualification for surgery. The data we present indicate that, throughout a wide range of cutoff points, elevated HbA1c is not an independent predisposing factor for postoperative complications in elective procedures of the hand and upper extremities. Given our findings and those of similar studies in the literature, we recommend that surgeons regard HbA1c as one piece of the surgical candidacy algorithm rather than an independent gatekeeper for elective procedures.
